# Tribological Behavior of Cotton Fabric/Phenolic Resin Laminated Composites Reinforced with Two-Dimensional Materials

**DOI:** 10.3390/polym15224454

**Published:** 2023-11-18

**Authors:** Yonggang Guo, Chenyang Fang, Tingmei Wang, Qihua Wang, Fuzhi Song, Chao Wang

**Affiliations:** 1 School of Mechanical and Electrical Engineering, Henan University of Technology, Zhengzhou 450001, China; nanogyg@163.com (Y.G.);; 2 State key Labratory of Solid Lubrication, Lanzhou Institute of Chemical Physics, Chinese Academy of Sciences, Lanzhou 730000, China; 3 Qingdao Center of Resource Chemistry & New Materials, Qingdao 266071, China

**Keywords:** two-dimensional materials, cotton fabric, phenolic resin, friction and wear, molecular dynamics

## Abstract

In this study, cotton fabric-reinforced phenolic resin (CPF) composites were modified by adding four two-dimensional fillers: graphitic carbon nitride (g-C_3_N_4_), graphite (Gr), molybdenum disulfide (MoS_2_), and hexagonal boron nitride (h-BN). The tribological properties of these modified materials were investigated under dry friction and water lubrication conditions. The CPF/Gr composite exhibits significantly better tribological performance than the other three filler-modified CPF composites under dry friction, with a 24% reduction in friction coefficient and a 78% reduction in wear rate compared to the unmodified CPF composite. Under water lubrication conditions, all four fillers did not significantly alter the friction coefficient of the CPF composites. However, except for an excessive amount of Gr, the other three fillers can reduce the wear rate. Particularly in the case of 10% MoS_2_ content, the wear rate decreased by 56%. Scanning electron microscopy (SEM) and X-ray photoelectron spectroscopy (XPS) were employed for the analysis of the morphology and composition of the transfer films. Additionally, molecular dynamics (MD) simulations were conducted to investigate the adsorption effects of CPF/Gr and CPF/MoS_2_ composites on the counterpart surface under both dry friction and water lubrication conditions. The difference in the adsorption capacity of CPF/Gr and CPF/MoS_2_ composites on the counterpart, as well as the resulting formation of transfer films, accounts for the variation in tribological behavior between CPF/Gr and CPF/MoS_2_ composites. By combining the lubrication properties of MoS_2_ and Gr under dry friction and water lubrication conditions and using them as co-fillers, we can achieve a synergistic lubrication effect.

## 1. Introduction

Oil-lubricated stern bearings have the risk of oil leakage and combustion in the process of use and are prone to wear and damage due to dry friction when the oil is cut off [1]. With growing environmental awareness and stricter pollution standards, people are urged to research and develop new bearing systems that are pollution-free, wear-resistant, efficient, energy-saving, long-lasting, and so on [2,3,4]. Water is the most promising lubricating medium due to its non-pollution, wide source, safety, and incombustibility properties [5,6]. However, there is a significant difference in the physical properties between water and lubricating oil [7]. Compared to lubricating oil, water has a low viscosity, making it difficult to form a lubricating film on the friction interface. Specifically, in frequent starting and stopping and variable load conditions, the lubrication between the shaft and the bearing is mixed lubrication, boundary lubrication, or dry friction conditions, which seriously reduces the safety and life of the stern bearing [8,9]. Therefore, it is very necessary to develop materials with excellent anti-friction and anti-wear properties in a water environment to address the limitations of water lubrication and improve the service life of bearings.

Since the last few decades, the tribology of polymeric materials has been attracting extensive interest from both academia and industry [10]. Polymeric materials are excellent candidates for tribo-materials for water lubrication conditions owing to their high chemical stability and self-lubrication. Examples include new-type nylon, modified rubbers, reinforced phenolic resin, and so on. High-performance fiber fabric-reinforced resin composites combine the advantages of both fibers and resins, making them a high-strength, low-self-lubricating bearing material [11,12,13]. It can be used in aviation, aerospace, shipbuilding, and so on. In the shipbuilding field, fabric resin laminated composites can be used as water-lubricated stern tube-bearing materials for ships using water as lubricant [14]. The fabric/resin interfacial affects the overall performance of the composites. Strong interfacial adhesion guarantees efficient stress transfer from the resin matrix to reinforced fibers and ensures the continued development of fiber-reinforced composites for potential advanced composite applications [15]. However, Nomex and Polytetrafluoroethylene (PTFE) PTFE fibers have low surface chemical activity, which reduces the bond strength between the fabric and the resin. Cotton fibers have moderate strength and better adhesion to resins than PTFE fibers and Nomex fibers. Meanwhile, as a commonly used material for water-lubricated bearings, the tribological properties of CPF composite directly affect the frictional wear, noise, operational stability, and service life of water-lubricated bearings. Therefore, it is of great significance to study the friction properties of CPF composites for water-lubricated bearings.

The properties of the resin matrix play a crucial role in the overall tribological performance of the composites [16]. Phenolic resin (PF) has the advantages of good mechanical properties, dimensional stability, a simple production process, and wear resistance, and is often used as an adhesive resin [17,18,19]. Despite the excellent properties of fiber fabric-reinforced PF composites, there are still some challenges in terms of friction and wear. This is manifested by the fact that pure PF often lacks self-lubricating properties, has a high coefficient of friction, significant friction noise, and poor dimensional stability when exposed to moisture [20,21]. Therefore, it is of great importance to improve the friction and wear properties of phenolic resins.

Modification of polymer resins by filler blending has become a common method to improve the tribological properties of fabric composites [22,23,24]. Expect nanoparticles, such as Si_3_N_4_, CaCO_3_, TiO_2_, CuO, Al_2_O_3_, ZnO, and SiC, to be filled into the PF matrix in order to improve the tribological and mechanical performance of PF composites. In recent years, two-dimensional materials such as g-C_3_N_4_, Gr, MoS_2,_ and h-BN have attracted interest from tribologists and engineers. More specifically, they have unique layered structures and low shear resistance, which endow them with excellent self-lubricating properties, due to which they can be widely used in the field of tribology [25,26]. The crystal structure of g-C_3_N_4_ has strong covalent C–N bonds and weak van der Waals forces between layers, which have excellent lubricity properties similar to those of carbon-based nanomaterials. Wu et al. [27] filled the prepared g-C_3_N_4_ nanosheets into a phenolic resin coating and investigated the effect on its tribological properties, suggesting that the modified tribological properties of g-C_3_N_4_ nanosheets may be attributed to their lamellar structure and high specific area, which contribute to the formation of a thin and continuous transfer film. Zhang et al. [28] investigated the influence of graphite, graphene, and graphene oxide fillers on the tribological performance of PTFE/Nomex hybrid fabric/phenolic resin composites and revealed that all three solid lubricant fillers were effective in reducing the wear rate of the fabric composites. Gao et al. [29] obtained significantly improved tribological performance by adding h-BN nanoparticles to a polyoxymethylene (POM) composite. MoS_2_ is an advanced lubricating material, but the low shear strength of MoS_2_ would be reduced in a humid environment [30]. However, more recent studies demonstrated that MoS_2_ could still maintain the capacity of lubrication in aqueous lubrication. Xin et al. [31] prepared MoS_2_/ polyether-ether-ketone (PEEK) PEEK composites using ball milling and spark plasma sintering processes, and the experimental results showed that MoS_2_/PEEK composites have excellent anti-wear properties under water lubrication conditions and are a potential material for underwater equipment.

Currently, there have been reports on the tribological properties of modified fiber fabric composites. However, most of these studies focus on PTFE fibers and Nomex fibers. There are fewer studies on the tribological behavior of CPF composites. Generally, solid lubricant filling can greatly improve the self-lubricating properties of the resin matrix, and at the same time, the resin with solid lubricant transferred to the counterpart can form a transfer film with lubricating properties. All these factors can promote the improvement of the anti-wear and friction-reduction properties of CPF composites. However, what types of two-dimensional materials are more suitable for water lubricating when modified to phenolic resin, and the reasons are unknown.

In order to explore the effects of different two-dimensional materials on the tribological behavior of cotton fabric/phenolic resin laminated composites, a series of CPF composites were modified by adding different proportions of four two-dimensional materials: g-C_3_N_4_, graphite (Gr), MoS_2_, and h-BN. The tribological properties of these modified materials were investigated under dry friction and water lubrication conditions. In order to better understand wear mechanisms, worn surfaces were analyzed by scanning electron microscopy (SEM) and X-ray photoelectron spectroscopy. Besides experimental investigations, a comprehensive understanding of the adsorption behaviors of CPF/Gr and CPF/MoS_2_ composites on counterpart surfaces at the molecular level was achieved by molecular dynamics simulations, respectively. The different friction and wear behaviors of CPF/Gr and CPF/MoS_2_ composites were attributed to the different surface adsorption energy and material transfer behaviors. It is expected that this study may be helpful to the application of the CPF composites to water-lubricated stern bearings.

## 2. Experimental

### 2.1. Materials

The pheolic resin was used as the adhesive. Cotton fabric is woven from cotton yarn. Molybdenum disulfide (MoS_2_, particle size 12–16 μm, ≥99.9%) was provided by Shanghai Macklin Biochemical Technology Co., Ltd. (Shanghai, China). Hexagonal boron nitride (h-BN, particle size 5–10 μm, ≥99.9%) was provided by Shanghai Macklin Biochemical Technology Co., Ltd. (China). Graphite (Gr, particle size 5–10 μm, ≥99%) was provided by Shanghai Meryer Chemical Technology Co., Ltd. (Shanghai, China). Anhydrous ethanol was provided by Qingdao SankaiMedical Technology Co., Ltd. (Qingdao, China). The g-C_3_N_4_ was synthesized by the calcination method, and the other chemical agents were used as received without further treatment. The four fillers were treated with ultrasonics before use.

The preparation process of the g-C_3_N_4_ is shown as follows [32]: Firstly, melamine powder was put into an alumina crucible with a lid, ramped at 10 °C/min to 550 °C, where it was held for 2 h, and then cooled to room temperature. The collected yellow products were dry rubbed in a ball mill for 12 h, and then the graphitic phase carbon nitride (g-C_3_N_4_) was obtained.

### 2.2. Sample Preparation

Firstly, the cotton fabric was cut into 70 cm × 20 cm for later use. The four fillers were added to the PF resin solution in the proportion shown in Table 1. The mixed solution was stirred through a stirrer (OS40-S, DLAB Scientific, Beijing, China)at 300 rpm for 1 h at room temperature to ensure the fillers were uniformly blended in the resin solution. Subsequently, the mixture is further processed with a three-roller calender to ensure even dispersion of the four fillers. After that, the dispersed resin solution was diluted to 50% with the appropriate amount of ethanol and mechanically stirred at 300 rpm for 1 h. Finally, the cotton fabric was submerged in the pre-prepared resin solution and subjected to ten minutes of ultrasonic treatment. After several cycles of immersion, the relative mass fraction of the resin mixture reached 60 ± 5%, and it was then placed in an oven at 110 °C for a three-minute drying period.

The impregnated cotton fabric was cut into 85 mm × 50 mm pieces, which were stacked layer by layer and then placed into the mold. The fabrication of CPF composites was carried out using a hot-pressing process, with curing at 2.6 MPa and 130 °C for 4 h. Finally, the CPF composites were taken out and cut into 25 mm × 10 mm × 3 mm to make the test samples.

### 2.3. Tribological Tests

**Figure 1:**
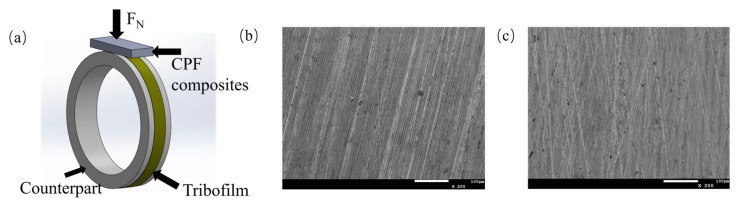
(**a**) Schematic diagram of block-on-ring wear tester; (**b**) SEM images of GCr15 surface; (**c**) SEM images of QSn7-0.2 surface.

Tribological tests were carried out using a block-on-ring apparatus (MRH-1A, Jinan Yihua Tribology Testing Technology Co., Ltd. (Jinan, China)). The structure of the friction pair is shown in Figure 1a. The test samples had a dimension of 25 × 10 × 3 mm^3^. GCr15 and QSn7-0.2 were selected as the counterparts under dry friction and water lubrication conditions, respectively. GCr15 steel is frequently used as bearing steel and has excellent mechanical properties but poor corrosion resistance in water lubrication, while QSn7-0.2 is used in the water environment due to its excellent corrosion resistance [33]. The diameter of the counterpart ring was 49.95 ± 0.05 mm. The surface of the ring was polished with SiC metallographic abrasive papers, and the mean roughness Ra was controlled at about 0.2 μm with randomly distributed grooves, as shown in Figure 1b,c. The counterpart ring was thoroughly cleaned with petroleum ether in an ultrasonic bath for 10 min before testing.

All the tribological tests were conducted at room temperature. For water lubrication, both the specimen and the counterpart were fully immersed in pure water. A constant normal load of 132 N was applied throughout the tests. The tribological experiments were performed at constant and variable sliding speeds, respectively. In the variable-speed friction experiment, the rotation speeds ranged from 35 to 1120 rpm, corresponding to velocities of 0.09 m/s, 0.18 m/s, 0.36 m/s, 0.73 m/s, 1.47 m/s, and 2.93 m/s. The speed was increased every 30 min, and the total duration of the experiment was 180 min. In the constant-speed friction experiment, the rotation speed was set at 140 rpm (0.36 m/s), and the experiment lasted for 120 min. Additionally, 132 N is equivalent to 0.25 Mpa in operation, and the variable sliding speed process simulates the different stages of bearing operation [34]. Every test was repeated three times, using the average value as the test result. The friction coefficient (COF) was automatically recorded by the tribometer. The specific wear rate of the test samples was calculated according to Equation (1) [35].

(1) $$W_{S}=\frac{B}{L\times F}\;[R^{2}\arcsin\left(\frac{W}{2R}\right)-\frac{W}{4}\sqrt{4R^{2}-W^{2}}]\cdot {\mathrm{mm}}^{2}/\mathrm{Nm}$$

where *B* is the width of polymer testing samples, *R* is the radius of the counterpart ring, *W* is the projected width of the wear track (depending on material loss), *F* is the normal load applied on polymer samples (132 N), and *L* is the total sliding distance (m).

### 2.4. Testing and Characterization

The particle size of the four fillers after ultrasonic treatment was measured using a laser particle size distribution meter (LF-POP, OmicronOMEC, Zhuhai, China). X-ray diffraction (XRD, Shimadzu 6000, Shimadzu, Kyoto, Japan) was used to observe the crystalline structures of the g-C_3_N_4_ powder with Cu–Ka radiation. The measurement speed was 10°min^−1^, and the measurement range was 10–80°. Fourier transform infrared spectroscopy (FTIR, TENSOR 27, Billerica, MA, USA) was used to observe the chemical structure composition of g-C_3_N_4_ using the KBr method in a wave number range of 4000–500 cm^−1^. A scanning electron microscope (SEM, JSM-7610F, Akishima-shi, Japan) was used to investigate the surface microscopy of the counterparts and the worn surface of fabric composites. X-ray photoelectron spectroscopy (XPS, Thermo Fisher Scientific, Waltham, MA, USA) with the line source of monochromatized Al Kα to analyze the changes in the phase composition and chemical composition of the worn surface of counterparts.

## 3. Results and Discussion

### 3.1. Characterization of g-C_3_N_4_ and Particle Size Distribution of Four Fillers

**Figure 2:**
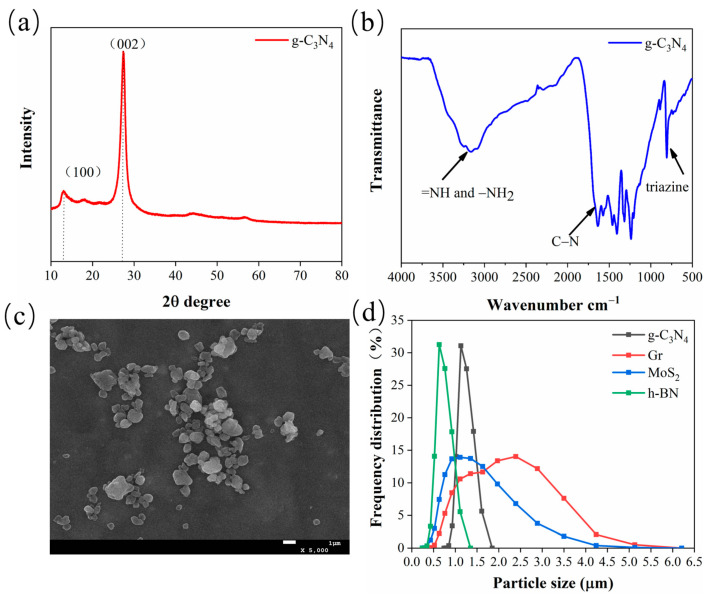
(**a**) XRD patterns of g-C_3_N_4_; (**b**) FTIR spectra of g-C_3_N_4_; (**c**) SEM micrograph of g-C_3_N_4_; (**d**) the particle size of the four fillers.

Figure 2a shows the g-C_3_N_4_ XRD pattern; it can be seen that two major diffraction peaks appear near 13.1° and 27.3°, and no diffraction peaks of other impurity phases were detected in all samples. The peaks located at 13.1° and 27.5° were attributed to the (100) and (002) planes, which were, respectively, related to the interlayer structural packing and interlayer stacking structure [25]. The molecular structure of g-C_3_N_4_ was further confirmed by FTIR spectroscopy, as shown in Figure 2b. The broad bands in the 3000–3500 cm^−1^ were attributed to the secondary (=NH) and primary (–NH_2_) amines. Noticeably, the set of peaks between 1200 cm^−1^ and 1700 cm^−1^ can be assigned to the typical stretching vibration of CN heterocycles. The distinct and sharp peak at around 807 cm^−1^ originated from representative tri-s-triazine units [27]. These observations confirm the successful synthesis of g-C_3_N_4_. Figure 2c shows the morphology of the synthesized g-C_3_N_4_ powder. It can be seen that g-C_3_N_4_ powder exhibits an irregular flake-like structure. Particle sizes of g-C_3_N_4_, Gr, MoS_2_, and h-BN after ultrasonic treatment used as filler to modify the CPF composites were tested by a laser particle size distribution instrument. As shown in Figure 2d, the particle sizes of these four fillers are similar, with an average particle size ranging from 0.7 to 2.5 μm.

### 3.2. Tribological Properties

#### 3.2.1. Tribological Behaviors at Varying Sliding Speeds under Water Lubrication

**Figure 3:**
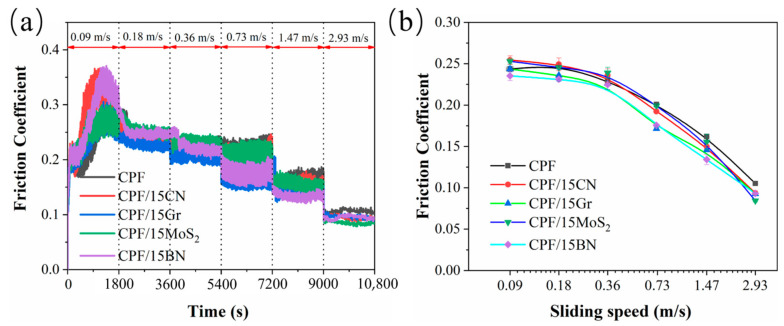
(**a**) Friction coefficients of CPF, CPF/15CN, CPF/15Gr, CPF/15MoS_2,_ and CPF/15BN as a function of sliding speed; (**b**) Stribeck curves derived from friction coefficients of various speed steps. Load: 132 N; lubrication medium: pure water.

In order to investigate the tribological performance of CPF composites under different lubrication regimes, tribological tests were performed at varying sliding speeds. Figure 3a shows the friction coefficient curve of unmodified and four types of two-dimensional material-modified CPF composites with a mass fraction of 15%. The applied load is 132 N, and the sliding speed increases from 0.09 m/s to 2.93 m/s, with a change in speed every 30 min. It can be seen that in the initial stages of friction, several CPF composites undergo a noticeable running-in process where the friction coefficient gradually increases and then stabilizes. As the velocity continues to increase, the friction coefficient of all CPF composites decreases at higher speeds. At lower speeds, compared to unmodified CPF composites, the friction coefficient of MoS_2_ and g-C_3_N_4_-modified CPF composites is relatively higher. However, when the velocity exceeds 0.36 m/s, the friction coefficient of these CPF composites significantly decreases, and it becomes lower than that of unmodified CPF composites.

The friction coefficient curves plotted based on the average values of friction coefficients within different velocity ranges are shown in Figure 3b. It can be observed that during the variable speed process, all five CPF composites exhibit typical Stribeck curve characteristics. This means that as the velocity increases, the friction system gradually transitions from boundary lubrication to mixed lubrication. However, throughout the entire process, the friction coefficients are noticeably higher than those under conventional water lubrication conditions. These results indicate that the friction interface is not purely in a state of either boundary lubrication or mixed lubrication; instead, it involves dry friction. This is mainly because the phenolic resin composites are relatively hard, and the contact load at the friction interface is significant, making them susceptible to puncturing the boundary adsorption film and experiencing dry friction, especially at low speeds [36]. Since 0.36 m/s marks the transition point for lubrication states, the subsequent study focuses on the friction and wear behavior of the CPF composites with four different filler contents under constant-speed conditions in both dry and water lubrication conditions.

#### 3.2.2. Tribological Behaviors at Constant Sliding Speeds

**Figure 4:**
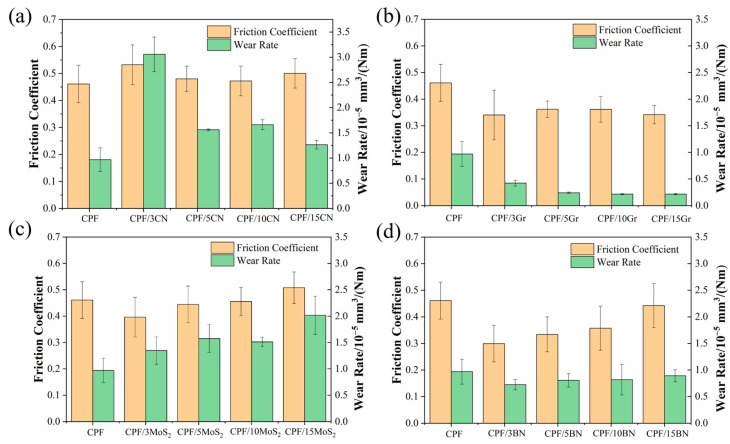
Friction coefficients and wear rates of CPF composites modified with different filler contents under dry friction conditions: (**a**) g-C_3_N_4_, (**b**) Gr, (**c**) MoS_2_, and (**d**) h-BN. Load: 132 N, rotational speed: 140 rpm.

Figure 4 shows the friction coefficients and wear rates of CPF composites modified with different filler contents under dry friction conditions. It can be seen from Figure 4a,c that the addition of g-C_3_N_4_ and MoS_2_ did not lead to an improvement in tribological performance, as the friction coefficients remained relatively unchanged and the wear resistance deteriorated. It is assumed that the harder g-C_3_N_4_ particles are transferred to the friction interface, causing “three-body” wear, resulting in a higher wear rate of the composites. At the same time, MoS_2_ tends to oxidize under dry friction conditions, reducing its own self-lubricating properties and giving it poor wear resistance [23]. In comparison, the incorporation of Gr can significantly reduce both the friction coefficients and wear rates of the CPF composites (Figure 4b). When the content increased to 10% and 15%, the friction coefficient decreased by 24%, and the wear rate decreased by 78%. As shown in Figure 4d, the addition of h-BN can significantly improve the tribological behavior of CPF composites. Even with a 3% h-BN content, a substantial 35% reduction in the friction coefficient and a 25% decrease in wear rates are achieved. Gr and h-BN have similar properties and excellent self-lubricating properties. Under dry friction conditions, it is easy to promote the transfer of wear debris to the counterpart surface, forming a continuous solid lubrication transfer film, which improves the tribological properties of the composites [28,37]. However, as the h-BN content increased further to 15%, a subtle trend of rising friction coefficients and wear rates emerged, resembling the behavior of unmodified CPF composites. Therefore, the CPF composites modified with 15% Gr and 3% h-BN have superior tribological properties under dry friction conditions. While the incorporation of g-C_3_N_4_ and MoS_2_ led to a corresponding increase in the friction coefficient and wear rate of the CPF composites, this result is consistent with the results of the friction coefficient at low speeds (Figure 3b), indicating that the CPF composites are accompanied by dry friction under low-speed boundary lubrication conditions.

**Figure 5:**
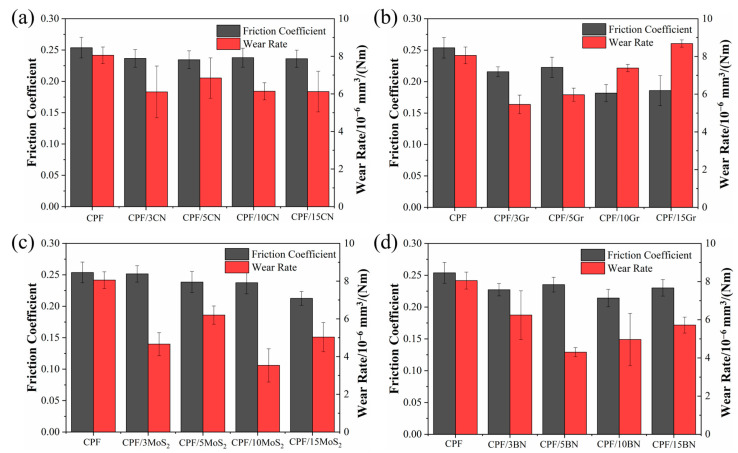
Friction coefficients and wear rates of CPF composites modified with different filler contents under water lubrication conditions: (**a**) g-C_3_N_4_, (**b**) Gr, (**c**) MoS_2_, and (**d**) h-BN. Load: 132 N; rotational speed: 140 rpm.

Figure 5 illustrates the friction coefficients and wear rates of CPF composites modified with varying filler contents under water lubrication with a load of 132 N and a speed of 140 rpm. The tribological properties of several composites, except CPF/Gr composites, showed significant improvement over dry friction conditions. The wear-reducing and anti-friction properties are attributed to the ability of tribofilms and boundary adsorption films to avoid direct solid-solid contact, thereby reducing friction and wear. It can be observed that the addition of all four two-dimensional materials leads to a decrease in the friction coefficient compared to unmodified CPF composites, but the changes with increasing content are not significant. By examining the wear rates, it becomes evident that the tribological performance of Gr under water lubrication conditions is notably lower than that under dry friction conditions. Furthermore, as the Gr content increases, the wear rate rises. When the content reaches 15%, the wear amount surpasses that of the unmodified CPF composite. This may be due to the presence of water, which hinders the formation of the transfer film of the CPF/Gr composite. Apart from Gr, the other three fillers can reduce the wear rate of the CPF composites, especially 5% h-BN and 10% MoS_2_, which exhibit the most significant reduction in wear amount. The wear rates are 4.3 × 10^−6^ mm^3^/Nm and 3.54 × 10^−6^ mm^3^/Nm, respectively, representing reductions of 47% and 56% compared to unmodified CPF composite. This may be attributed to the fact that h-BN reacted with water molecules to generate B_2_O_3_ and H_3_BO_3_ [29], and MoS_2_ could still maintain the capacity of lubrication in aqueous lubrication [31]. They generate a transfer film with a certain load-bearing capacity, which improves the wear resistance of the composites. The analysis of friction coefficients and wear rates among the several CPF composites highlights substantial differences in the tribological performance of Gr and MoS_2_ in different environments.

**Figure 6:**
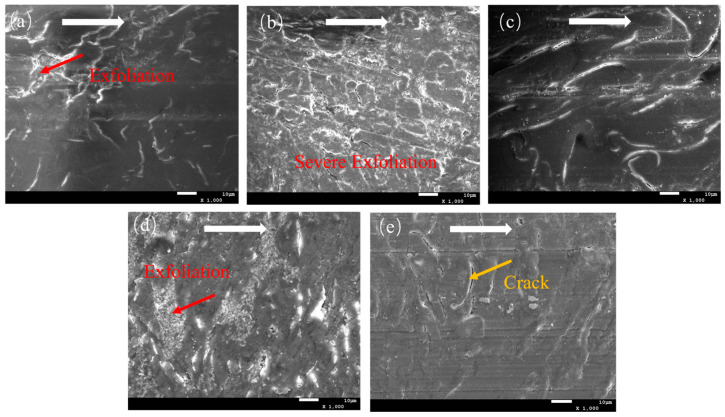
SEM images of worn surfaces of (**a**) CPF, (**b**) CPF/15CN, (**c**) CPF/15Gr, (**d**) CPF/15MoS_2_, and (**e**) CPF/15BN under dry friction conditions at 132 N and 140 rpm. White arrows indicate the sliding direction.

The SEM images of the wear surfaces of five CPF composites after dry friction are shown in Figure 6. It can be seen that the wear surface of unmodified CPF composite exhibits slight resin delamination than the unworn surface (Figure S1a), with the primary wear mechanism being fatigue wear. After adding g-C_3_N_4_ and MoS_2_, as shown in Figure 6b,d, the composites experience significant resin delamination, accompanied by numerous furrows or grooves. The pristine CPF composites had no visible flaking on the surface and relatively smooth surfaces, as shown in Figure S1b,d. The wear debris consists of severed cotton fibers and resin fragments, illustrating a typical abrasive wear mechanism. In contrast, when Gr is introduced, the wear surface is smooth with fewer cracks compared to the original surface (Figure S1c)., and there is no apparent resin delamination. It can be attributed to the excellent lubricating properties of Gr [38], as it can transfer to the friction interface during dry friction, leading to friction reduction and high wear resistance. After the addition of h-BN, only a small number of fatigue cracks appear on the wear surface (Figure S1e), and there is no pronounced resin delamination. The wear performance of the composites is improved compared to that with g-C_3_N_4_ and MoS_2_, but it is inferior to the wear resistance of the CPF composite with Gr added.

**Figure 7:**
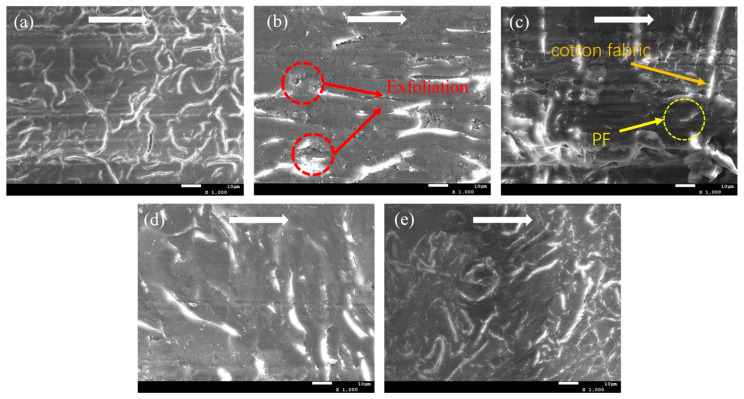
SEM images of worn surfaces of (**a**) CPF, (**b**) CPF/15CN, (**c**) CPF/15Gr, (**d**) CPF/15MoS_2_, and (**e**) CPF/15BN under water lubrication conditions at 132 N and 140 rpm. White arrows indicate the sliding direction.

Figure 7 demonstrates the SEM images of the worn surfaces of five types of CPF composites under water-lubricating conditions. Due to the poor water resistance of phenolic resin, water penetrates into the interior of the composites, resulting in some microcracks on the surface of the CPF composites (c.f. Figure S2) [39]. As shown in Figure 7a of the unmodified CPF composite, although the surface is smooth, there are many microcracks present. During the frictional shear process, they damage the resin/cotton fiber interface, causing it to detach and ultimately develop into cracks. The addition of g-C_3_N_4_ results in a significant reduction in cracks on the worn surface, but resin delamination similar to dry friction conditions still exists (Figure 7b). After the addition of h-BN, the worn surface shows no significant difference from the unmodified CPF composite except for the reduction in surface cracks (Figure 7e).

It should be noted that the addition of Gr and MoS_2_ exhibits a completely different wear morphology from dry friction conditions. The CPF composite modified with Gr does not show a smooth surface, similar to dry friction conditions. Instead, there is a significant amount of resin delamination and cotton fibers exposed on the surface. During the friction process, cotton fibers are cut and pulled out, leading to the overall structural damage of the composites (Figure 7c). After the addition of MoS_2_, the worn surface is smooth, with no obvious cotton fibers exposed on the surface. Resin and cotton fibers jointly withstand the shear stress during the friction process, demonstrating good wear resistance. The wear morphology results are consistent with the wear rate results in Figure 5.

#### 3.2.3. Tribo-Chemistry of Counterpart Surface with Addition of Gr and MoS_2_

From the above results, it can be seen that Gr and MoS_2_-modified CPF composites exhibit significant differences in friction and wear performance. CPF/15Gr composite show excellent anti-friction and wear properties under dry friction conditions but have relatively poor wear resistance under wet lubrication conditions. On the other hand, the CPF/15MoS_2_ composite demonstrates some wear resistance under wet lubrication conditions while lacking anti-friction and wear performance under dry friction conditions. In the following, we will focus on the performance differences between the two and analyze their interfacial transfer film morphology and chemical composition, with the aim of revealing their friction and wear mechanisms.

**Figure 8:**
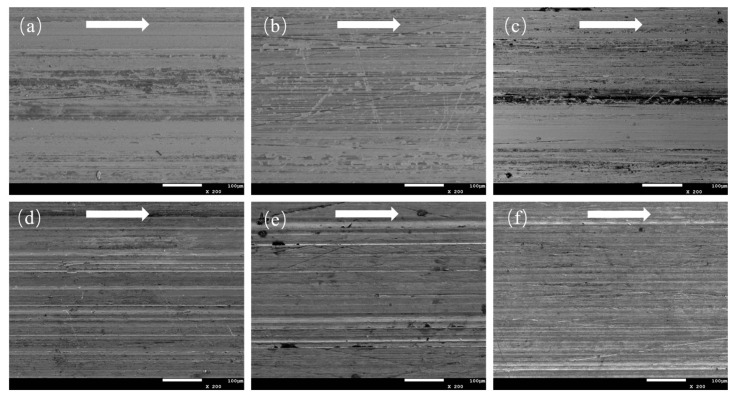
SEM images of the counterpart surfaces of (**a**,**d**) CPF, (**b**,**e**) CPF/15Gr, and (**c**,**f**) CPF/15MoS_2_ at 132 N and 140 rpm; (**a**–**c**) under dry friction conditions, (**d**–**f**) under water lubrication conditions. White arrows indicate the sliding direction.

Figure 8 shows SEM images of the counterpart surface morphology after sliding against unmodified CPF, CPF/15Gr, and CPF/15MoS_2_ composites under dry friction and water lubrication conditions. It can be seen that although a transfer film is formed for the unmodified CPF composite under dry friction, it is thick and discontinuous. This implies that the adhesion of the formed transfer film to the counterpart is relatively weak, making it easily removed during the friction process, leading to a higher wear rate (Figure 8a). After the addition of Gr, the worn surface is relatively smooth, with no apparent resin delamination or fiber exposure (Figure 6c). The formed transfer film is thin and uniform, exhibiting good adhesion to the couple. This prevents direct contact between the couple and the polymer during friction, thereby improving the material’s wear resistance (Figure 8b). As shown in Figure 8c, when MoS_2_ is added as a filler, severe scratching occurs on the counterpart surface, resulting in deep grooves and high surface roughness, increasing the wear of the composites.

Under water lubrication conditions, as shown in Figure 8d,e, the worn surfaces of the QSn7-0.2 counterpart after sliding against CPF/15Gr and CPF/15MoS_2_ composites exhibit completely different morphologies compared to dry friction. The CPF/15Gr composite counterpart surface shows numerous furrows caused by cotton fibers without a uniform and dense transfer film formation, as shown in Figure 8e. In contrast, the CPF/15MoS_2_ composite counterpart surface has a smooth, worn surface with a noticeable presence of transfer film. The worn surface of the unmodified CPF composite counterpart surface is similar to that of the CPF/15Gr composite counterpart, also showing a large number of furrows, indicating a similar wear mechanism between the two.

**Figure 9:**
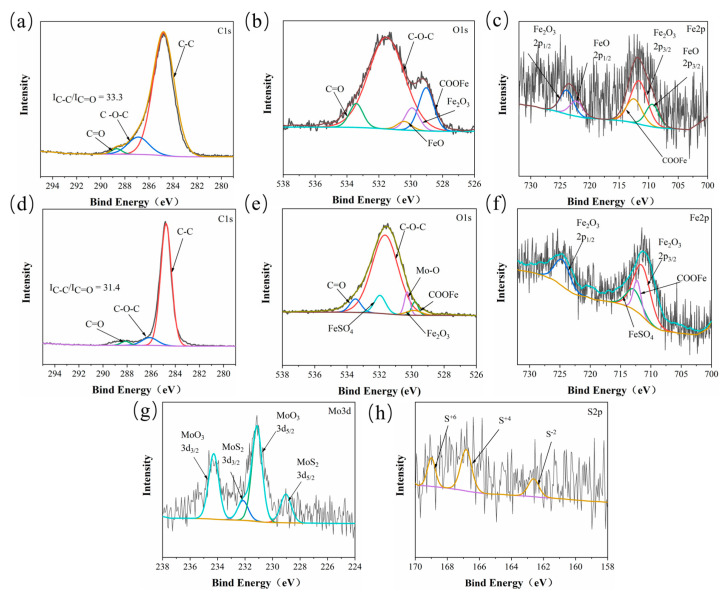
XPS spectra of CPF/15Gr ((**a**): C1s, (**b**): O1s, (**c**): Fe2p) and CPF/15MoS_2_ ((**d**): C1s, (**e**): O1s, (**f**): Fe2p, (**g**): Mo3d, and (**h**): S2p) tribofilms on the worn surface of GCr15 with CPF composites after sliding against under dry friction conditions at 132 N, 140 rpm.

To gain a deeper understanding of the mechanism of frictional chemical reactions, Figure 9 shows the XPS spectrum of the counterpart surfaces after sliding against the CPF/15Gr and CPF/15MoS_2_ composites under dry friction conditions. It can be seen that the counterpart surfaces of Gr and MoS_2_ exhibit similar C1s spectra. Among them, the feature peak corresponding to 284.6 eV is attributed to C–C bonds, mainly originating from PF, Gr, and cotton fibers. The peak at 286.9 eV corresponds to C–O–C bonds, primarily originating from PF and cotton fibers, while the peak at 288.2 eV corresponds to C=O bonds, originating from cotton fibers (Figure 9a,d). By calculating I_C–C_/I_C=O_, it can be seen that the I_C–C_/I_C=O_ ratio on the CPF/15Gr composite counterpart surface is higher than that of the CPF/15MoS_2_ composite counterpart surface, indicating the involvement of Gr in the construction of the transfer film on the interface [40,41]. In the Mo3d spectrum, strong peaks appear at 231.2 eV and 232 eV, corresponding to MoO_3_, suggesting that MoS_2_ is oxidized into MoO_3_ during friction, resulting in the loss of the self-lubricating properties of MOS_2_ [42]. This has an adverse effect on the friction and wear performance of the CPF composites, leading to higher friction coefficients and wear rates (Figure 9e,g).

Comparing the O1s spectra of the two, a strong peak at 529.1 eV, corresponding to the presence of organometallic compounds, appeared on the CPF/15Gr composite counterpart surface. Due to shear forces and frictional heat, molecular chain breakage occurs in the phenolic resin and cotton fibers, resulting in the formation of polymer carbon radicals. These carbon radicals can react with O_2_ and H_2_O in the air and ultimately react with the Fe present in the coupling, leading to the generation of organometallic compounds [43]. The formation of organometallic compounds is beneficial for enhancing the bond strength between the transfer film and the coupled surface, thereby better utilizing their lubrication and wear-resistant properties [40]. It can be seen from Figure 9e that a significant amount of Mo is oxidized into MoO_3_, and S elements can also react with the Fe in the coupling, generating FeSO_4_ (Figure 9e). It should be noted that the presence of reactive Mo and S elements inhibits the occurrence of chelation reactions, leading to the low intensity of organometallic compounds.

**Figure 10:**
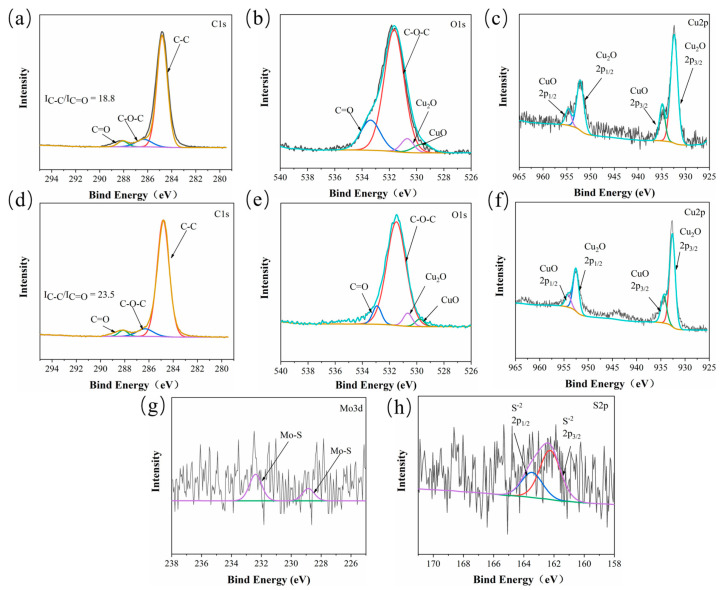
XPS spectra of CPF/15Gr ((**a**): C1s, (**b**): O1s, (**c**): Fe2p) and CPF/15MoS_2_ ((**d**): C1s, (**e**): O1s, (**f**): Fe2p, (**g**): Mo3d, and (**h**): S2p) tribofilms on the worn surface of QSn7-0.2 with CPF composites after sliding against under water lubrication conditions at 132 N, 140 rpm.

XPS spectra of the counterpart surface after sliding against the CPF/15Gr and CPF/MoS_2_ composites under water lubrication conditions are shown in Figure 10. Similar to Figure 9, the carbon peak is almost the same. However, the intensity of I_C–C_/I_C=O_, especially for CPF/15Gr composite, is significantly lower compared to dry friction conditions, indicating the ability to form a transfer film on the friction interface is weaker in the presence of water. Observing the O1s spectra of both, in contrast to dry friction, there is no evidence of chelation compound formation on the counterpart, indicating that under water lubrication conditions, the necessary conditions for chemical reactions seen in dry friction are not present. This leads to weaker adhesion between the transfer film and its counterpart. Furthermore, it can be seen that characteristic peaks corresponding to Mo–S at 228.9 eV and 232.7 eV in the Mo3d spectrum, as well as the corresponding feature peak S^−2^ at 162.4 eV in the S2p spectrum, indicate that under water lubrication conditions, MoS_2_ has not undergone oxidation, thereby preserving its self-lubricating properties. Therefore, the CPF/15MoS_2_ composite exhibits better tribological performance compared to the CPF/15Gr composite under water lubrication conditions.

#### 3.2.4. Molecular Dynamics Simulation of CPF Composites Modified by Gr and MoS_2_

**Figure 11:**
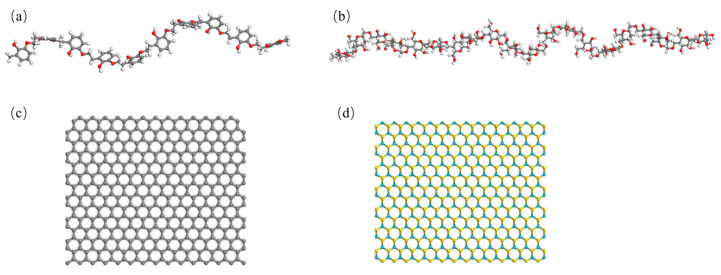
Molecular models of (**a**) PF, (**b**) cotton fabric, (**c**) Gr, and (**d**) MoS_2_.

Based on the above analysis, the differences in the tribological performance of CPF/Gr and CPF/MoS_2_ composites under dry friction and water lubrication conditions primarily stem from the formation of high-performance transfer films. The adsorption capability between the material and its counterpart directly influences the material’s transfer [44]. In the following, molecular dynamics simulations were used to investigate the adsorption behavior of CPF/Gr and CPF/MoS_2_ composites on their counterparts under both air and water environments. It can better explain the difference in tribological properties of the two materials in different environments. The main components of GCr15 and QSn7-0.2 are Fe and Cu, so Fe and Cu were chosen as the friction layers of the molecular models of their counterparts [45]. The model system consists of PF, cotton fabric, Gr, and MoS_2_, as shown in Figure 11. In addition, the gray balls, white balls, red balls, yellow balls, and blue balls represent carbon atoms, hydrogen atoms, oxygen atoms, sulfur atoms, and molybdenum atoms. Molecular models of CPF composites were constructed via the amorphous cell packing task in Materials Studio software(8.0), as shown in Figure S3.

**Figure 12:**
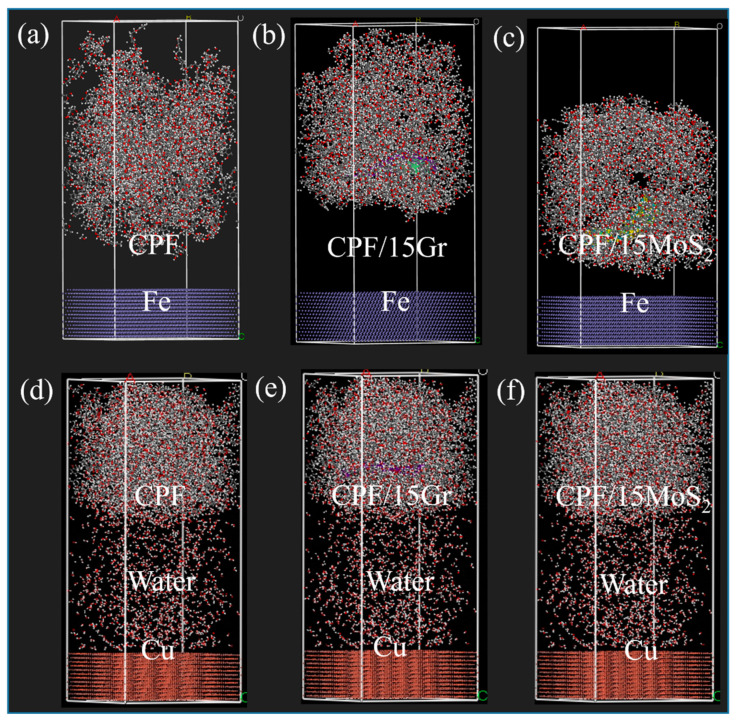
CPF composites adsorption model under different lubrication conditions: (**a**–**c**) under dry friction conditions and (**d**–**f**) under water lubrication conditions.

The amorphous cell module was used to construct the amorphous cell of the CPF composites, and it was optimized, as shown in Figure 12. The calculations were performed using the NVT ensemble with zero total momentum, the universal force field, the Nose temperature control method to maintain the temperature at 300 K (simulating room temperature), a van der Waals force cutoff radius of 12.5 Å (1 Å = 0.1 nm), and a Coulomb force accuracy of 0.01 Kcal·mol^−1^. The time step for calculations was set to 0.5 fs, and the simulation time was 100 ps. Adsorption energy calculations were then performed on the optimized model using the following formula [46]:

E = −E_inter_ = E_total_ − (E_layer1_ − E_layer2_) (2)

where E is the adsorption energy, E_inter_ is the interaction energy between the CPF composite and counterpart surface, E_layer1_ is the interaction between the CPF composite, and E_layer2_ is the interaction energy of the counterpart surface, in Kcal/mol.

The adsorption energy of the optimized model is calculated as shown in Table 2 and Table 3.

It can be seen that there is adsorption between the CPF composites and their counterparts. Under dry friction conditions, the CPF/15Gr composite exhibits higher adsorption energy. This suggests that under dry friction conditions, the adsorption between the CPF/15Gr composite and the Fe counterpart further promotes the formation and stable presence of the transfer film. This prevents direct contact at the friction interface, leading to effective lubrication and wear resistance. Under water lubrication conditions, the adsorption energies between CPF/15MoS_2_ and CPF composite have increased, while the adsorption energy of CPF/15Gr is lower than that under dry friction conditions. This indicates that the adsorption capacity of CPF/15Gr to the counterpart is weaker under water lubrication conditions, which is not beneficial to the formation of a transfer film. As a result, the tribological performance decreases compared to dry friction conditions.

#### 3.2.5. The Synergistic Interaction between MoS_2_ and Gr

The earlier analysis indicates that CPF/Gr and CPF/MoS_2_ composites are suitable for working under dry friction and water lubrication conditions, respectively. The reason for this lies in whether a self-lubricating transfer film has formed on the friction interface. To verify if there is good synergistic lubrication between the two fillers, a design was proposed to add both fillers together to prepare corresponding CPF composites. Subsequently, the friction coefficients and wear rates were tested under dry friction and water lubrication conditions.

**Figure 13:**
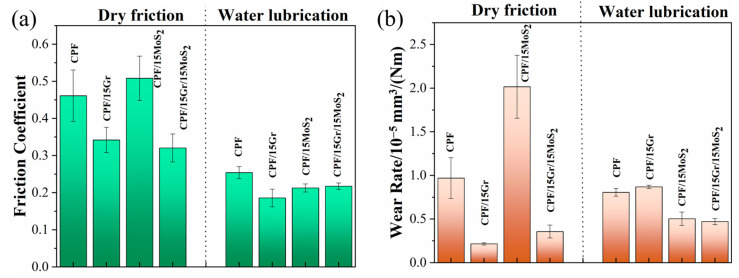
(**a**) Friction coefficients; (**b**) wear rates of CPF, CPF/15Gr, CPF/15MoS_2,_ and CPF/15Gr/15MoS_2_ at 132 N and 140 rpm.

As shown in Figure 13, the CPF/15Gr/15MoS_2_ composite exhibits good tribological performance under both dry friction and water lubrication conditions. Under dry friction conditions, there is a 30% reduction in the friction coefficient and a 63% decrease in the wear rate when compared to unmodified CPF composite. Under water lubrication conditions, the friction coefficient decreases by 16%, and the wear rate decreases by 41%. The combination of both fillers preserves the excellent tribological performance of Gr under dry friction conditions and maintains the self-lubricating properties of MoS_2_ under water lubrication conditions. This demonstrates that MoS_2_ and Gr have excellent synergistic lubrication effects.

## 4. Conclusions

The tribological performance of CPF composites modified with g-C_3_N_4_, Gr, MoS_2,_ and h-BN under both dry friction and water lubrication conditions was studied. The main conclusions are as follows:

1. Gr exhibits significantly better tribological performance than the other three fillers under dry friction. When the Gr content increased to 10% and 15%, the friction coefficient decreased by 24%, and the wear rate decreased by 78% compared to the unmodified CPF composite. The enhanced wear resistance can be attributed to promoting the transfer of wear debris to the counterpart surface, forming a continuous solid lubrication transfer film that improves the tribological properties of the composites.
2. Under water lubrication conditions, all four fillers did not significantly alter the friction coefficient of the CPF composites. However, except for an excessive amount of Gr, the other three fillers can reduce the wear rate of the composite material. Particularly in the case of 5% h-BN and 10% MoS_2_, the wear rate decreased by 47% and 56%, respectively. It should be noted that 10wt% MoS_2_ has the best wear-reduction effect. It demonstrated that certain content of MoS_2_ has not undergone oxidation, thereby preserving its self-lubricating properties. However, due to the presence of water, CPF/MoS_2_ composites are more easily transferred to the counterpart surface to form a transfer film with lubricating properties than CPF/Gr composites.
3. The adsorption capacity between the composites and their counterparts plays a crucial role in the formation of the transfer film under both dry friction and water lubrication conditions. Calculations of adsorption energy reveal that Gr is well-suited for dry friction, while MoS_2_ is more suitable for operation in a water environment. Using both MoS_2_ and Gr as co-modifiers for CPF composites, it can be found that the CPF composites have good tribological properties both under dry friction and water lubrication conditions. The results show that Gr and MoS_2_ have synergistic lubrication effects.

## Figures and Tables

**Table 1:** Designations and detailed compositions (wt%) of CPF composites.

Composites/wt%	PF	g-C_3_N_4_	Gr	MoS_2_	h-BN
CPF	100	/	/	/	/
CPF/3CN	97	3	/	/	/
CPF/5CN	95	5	/	/	/
CPF/10CN	90	10	/	/	/
CPF/15CN	85	15	/	/	/
CPF/3Gr	97	/	3	/	/
CPF/5Gr	95	/	5	/	/
CPF/10Gr	90	/	10	/	/
CPF/15Gr	85	/	15	/	/
CPF/3MoS_2_	97	/	/	3	/
CPF/5MoS_2_	95	/	/	5	/
CPF/10MoS_2_	90	/	/	10	/
CPF/15MoS_2_	85	/	/	15	/
CPF/3BN	97	/	/	/	3
CPF/5BN	95	/	/	/	5
CPF/10BN	90	/	/	/	10
CPF/15BN	85	/	/	/	15

**Table 2:** Adsorption energy of three CPF composites under dry friction conditions.

	E_total_	E_layer1_	E_layer2_	E
CPF	22,063.98	22,129.34	−42.6215	22.7391
CPF/15Gr	59,974.35	60,044.69	−42.6215	27.7161
CPF/15MoS_2_	38,583.75	38,650.52	−42.6215	24.1535

**Table 3:** Adsorption energy of three CPF composites under water lubrication conditions.

	E_total_	E_layer1_	E_layer2_	E
CPF	24,527.14	20,324.73	4227.439	25.0309
CPF/15Gr	52,501.46	48,298.61	4227.439	24.589
CPF/15MoS_2_	44,046.73	39,845.03	4227.439	25.7347

## Data Availability

The data that support the findings of this study are available upon request from the corresponding author.

## Supplementary Materials

The following supporting information can be downloaded at: https://www.mdpi.com/article/10.3390/polym15224454/s1, Figure S1: SEM images of worn surfaces of (a) CPF, (b) CPF/15CN, (c) CPF/15Gr, (d) CPF/15MoS_2_, (e) CPF/15BN under dry friction conditions. Figure S2: SEM images of worn surfaces of (a) CPF, (b) CPF/15CN, (c) CPF/15Gr, (d) CPF/15MoS_2_, (e) CPF/15BN under dry friction conditions. Figure S3. The molecular structures of (a) CPF, (b) CPF/15Gr and (c) CPF/15MoS_2_.
