# Supplementary material for: Tribological Behavior of Cotton Fabric/Phenolic Resin Laminated Composites Reinforced with Two-Dimensional Materials
Source: Polymers (Basel). 2023 Nov 18;15(22):4454. doi: 10.3390/polym15224454 (PMC10675720; doi:10.3390/polym15224454)
Supplement: Supplementary file 1 [file polymers-15-04454-s001.zip › polymers-2696798-supplementary.pdf]

Supplementary Material

Tribological Behavior of Cotton Fabric/Phenolic Resin Laminated Composites Reinforced with Two-Dimensional Materials

Yonggang Guo [1], Chenyang Fang [1], Tingmei Wang [2], Qihua Wang [2], Fuzhi Song [2,3,*] and Chao Wang [2,3,*]

[1] School of Mechanical and Electrical Engineering, Henan University of Technology, Zhengzhou 450001, China; nanogyg@163.com (Y.G.)
[2] State key Labratory of Solid Lubrication, Lanzhou Institute of Chemical Physics, Chinese Academy of Sciences, 730000. Lanzhou, China; wangtin3088@sina.com (T.W.); wangqh@licp.cas.cn (Q.W.)
[3] Qingdao Center of Resource Chemistry & New Materials, Qingdao, 266071, China
* Correspondence: fuzsong@licp.cas.cn (F.S.); wangc@licp.cas.cn (C.W.)

1. SEM images of before the tests

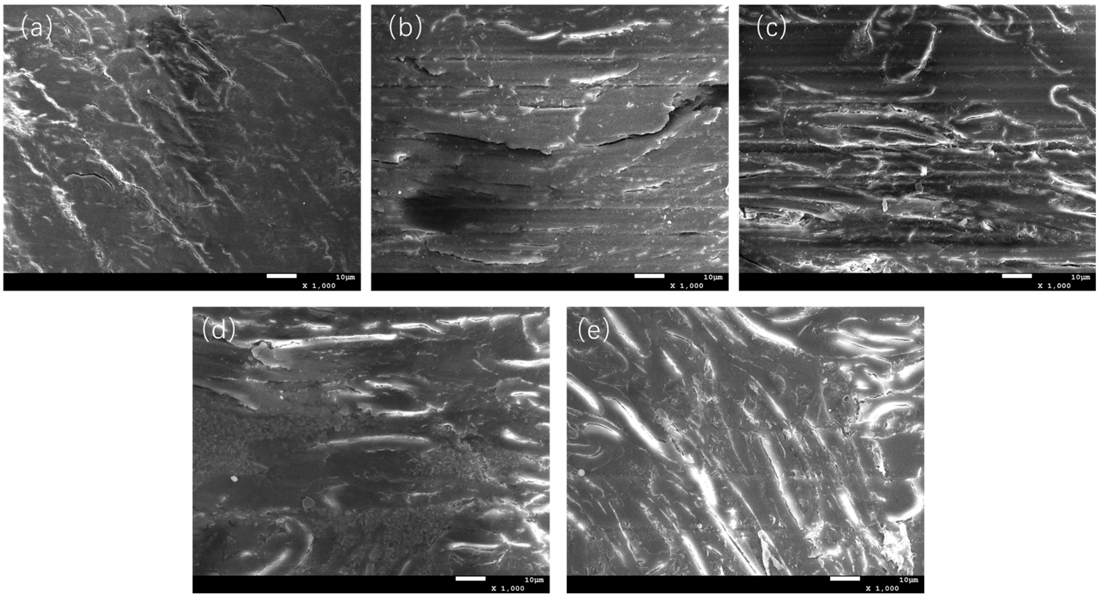

Figure.S1 SEM images of worn surfaces of (**a**) CPF, (**b**) CPF/15CN, (**c**) CPF/15Gr, (**d**) CPF/15MoS_2, (**e**) CPF/15BN under dry friction conditions.

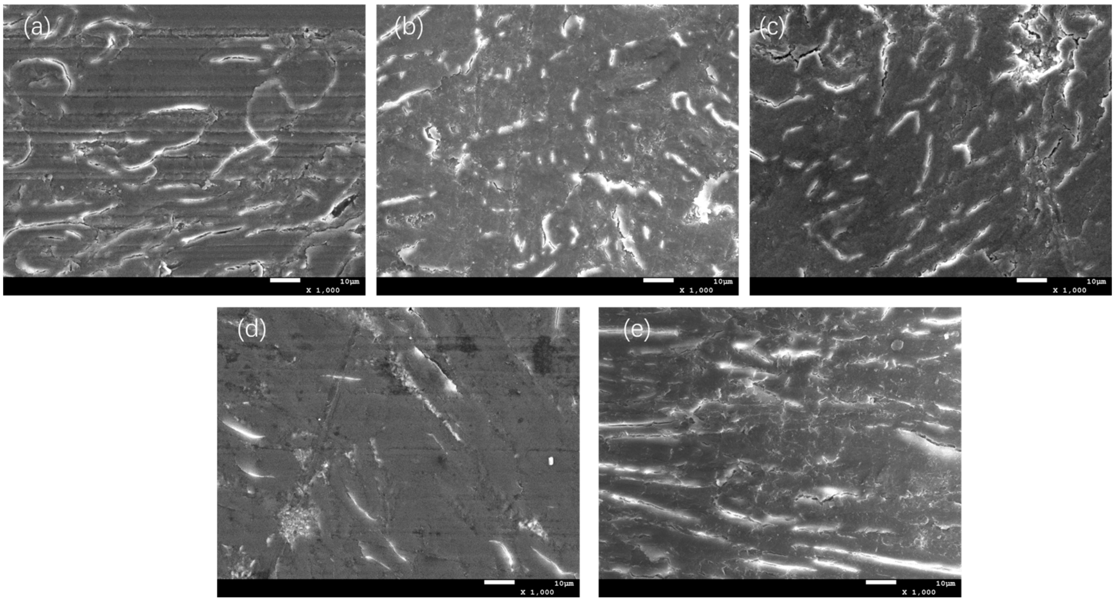

Figure.S2 SEM images of worn surfaces of (**a**) CPF, (**b**) CPF/15CN, (**c**) CPF/15Gr, (**d**) CPF/15MoS_2, (**e**) CPF/15BN under dry friction conditions.

2.Simulation methodology.

In the process of model building, Amorphous Cell Packing task in Materials Studio software was applied to build molecular models of CPF, CPF/15Gr and CPF/15MoS_2. Ewald method and atom-based method were utilized to analyze Coulomb interaction and van der Waals interaction. Force field used in this work was universal force-field that was the universally applicable force fields. Universal force fields are designed to be able to simulate many types of molecules and materials, not just limited to specific molecular species. In the modeling of CPF composites as shown in Fig.S1, corresponding molecular chains of cotton fabric, Gr, MoS_2 and phenolic resin were randomly packed in periodic boundary cells on the basis of Monte Carlo method. In addition, the gray balls, white balls, red balls, yellow balls, and blue balls represent carbon atoms, hydrogen atoms, oxygen atoms, sulfur atoms, and molybdenum atoms. Adsorption model was a double-layer molecular structure containing polymeric and top metal layers with a size of 5.4 nm × 5.4 nm × 12.9 nm (1 Å = 0.1 nm).

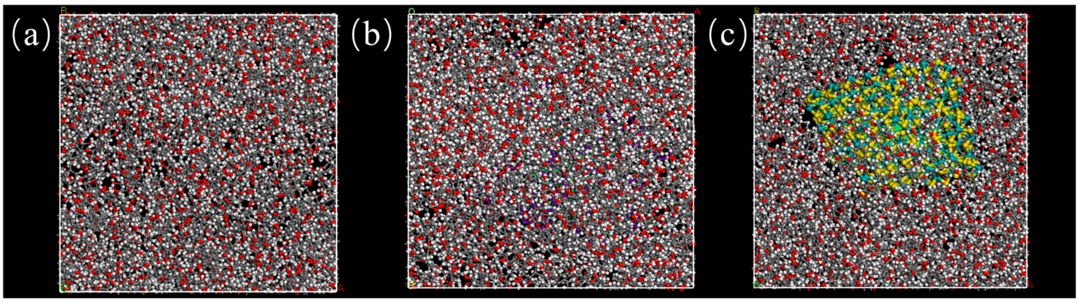

Figure.S3 The molecular structures of (**a**) CPF, (**b**) CPF/15Gr and (**c**) CPF/15MoS_2.

After the molecular models were constructed, the structure of the entire system was in an extremely instable. Therefore, the next work was to find the most stable structure through geometric optimization. It should be pointed out that annealing process and dynamic balance were performed by Forcite module for well-equilibration configurations. Local minimum energy state was obtained by virtue of a geometry optimization using smart algorithm with an energy convergence criterion of 0.00001 -Kcal/mol and force convergence criteria of 0.0001 Kcal/mol. Subsequently, the models were further equilibrated by a 5-cycle anneal process which was implemented under constant-temperature and constant-volume (NVT ensemble) with temperature ranging from 300 K to 600 K for relaxing the structure to obtain a stable state. And then, the configurations were dynamically balanced using NVT ensemble in a time of 0.5 fs. Energy was realized by energy module and the data is automatically exported in status.txt.
